# Towards Predicting Patient-Specific Flow-Diverter Treatment Outcomes for Bifurcation Aneurysms: From Implantation Rehearsal to Virtual Angiograms

**DOI:** 10.1007/s10439-015-1395-3

**Published:** 2015-08-04

**Authors:** T. W. Peach, K. Spranger, Y. Ventikos

**Affiliations:** Department of Mechanical Engineering, University College London, London, UK; Department of Engineering Science, University of Oxford, Oxford, UK

**Keywords:** Bifurcation aneurysm, Flow-diverter, Stent, Computational fluid dynamics, Virtual deployment, Virtual contrast

## Abstract

Despite accounting for the majority of all cerebral aneurysm cases, bifurcation aneurysms present many challenges to standard endovascular treatment techniques. This study examines the treatment of bifurcation aneurysms endovascularly with flow-diverting stents and presents an integrative computational modeling suite allowing for rehearsing all aspects of the treatment. Six bifurcation aneurysms are virtually treated with 70% porosity flow-diverters. Substantial reduction (>50%) in aneurysm inflow due to device deployment is predicted in addition to reductions in peak and average aneurysm wall shear stress to values considered physiologically normal. The subsequent impact of flow-diverter deployment on daughter vessels that are jailed by the device is investigated further, with a number of simulations conducted with increased outlet pressure conditions at jailed vessels. Increased outlet pressures at jailed daughter vessels are found to have little effect on device-induced aneurysm inflow reduction, but large variation (13–86%) is seen in the resulting reduction in daughter vessel flow rate. Finally, we propose a potentially powerful approach for validation of such models, by introducing an angiographic contrast model, with contrast transport modeled both before and after virtual treatment. Virtual angiograms and contrast residence curves are created, which offer unique clinical relevance and the potential for future *in vivo* verification of simulated results.

## Introduction

Over half (57.6%) of all cerebral aneurysms are found to occur at vessel bifurcations.^1^ The treatment of bifurcation aneurysms that are at risk of rupture is considered significantly more problematic than for simpler sidewall cases.^19^ Current treatment of bifurcation aneurysms is dominated by coiling for narrow-necked cases and stent-assisted coiling, where a scaffold in the vessel lumen is needed to prevent coil prolapse in more wide-necked geometries. Such stent-assisted procedures are highly complex and often require ‘Y-stenting,’ whereby a stent is deployed in two daughter vessels, and the proximal end of one stent protrudes into the second stent, creating a single stent lumen in the parent vessel.^11^

Bifurcation aneurysms, and wide-necked cases in particular, may be amenable to treatment by flow-diverter.^3^,^26^ Practically, treatment by flow-diverter must provide sufficient resistance to flow entering the aneurysm so as to ensure occlusion of the aneurysm dome by thrombosis. The high aneurysm inflow rates and substantial jetting often seen in bifurcation cases render successful aneurysm occlusion more elusive than in sidewall cases. Additionally, this flow resistance must be achieved without risking the complete occlusion of the non-stented daughter vessel in the bifurcation, which is jailed by the device. In short, the device must simultaneously restrict and promote flow in areas of the vasculature that lie adjacent to one another.^19^ This unique, and perhaps unrealistic, performance requirement has resulted in significant concerns being expressed in the literature regarding the efficacy of flow-diverter treatment in bifurcation aneurysms.^14^,^39^

Animal models have suggested that the effectiveness of flow-diverter treatment for inducing aneurysm occlusion in bifurcation cases may be substantially reduced. One canine study reported aneurysm occlusion at 3-months follow-up in only 14% of cases, although any sidewall branches jailed by the flow-diverter remained fully patent at follow-up in all cases.^7^ In a similar canine model, Raymond *et al.* showed incomplete aneurysm occlusion at 3-months follow-up in all experimental bifurcation aneurysms that were implanted with a flow-diverter. Reduction in aneurysm size was seen in around half of the geometries, but such a reduction was also accompanied by daughter vessel stenosis.^23^

Additionally, significant concerns of jailed vessel occlusion have been reported for clinical cases in the literature. A study conducted by Saatchi *et al.* included 46 ‘uncoilable’ aneurysms that originated at vessel bifurcations, with successful treatment by the *Pipeline*™ *Embolization Device* (PED) reported in 41 cases.^26^ In the five remaining cases (10.9% of the total), complete occlusion of the jailed daughter vessel was reported, which contributed to the death of one patient. Similar results were seen by Saleme *et al.* in a retrospective analysis of 37 bifurcation aneurysms treated by flow-diverter.^29^ Complete aneurysm occlusion at 6-months follow-up was reported in 97.3% of cases, but new permanent neurological deficit was seen in 9.4% of patients. Jailed daughter vessel occlusion at follow-up was seen in 32.4% of aneurysms, and visible vessel narrowing was evident in a further 40.5% of cases. However, the effects of vessel occlusion or narrowing were only symptomatic in 13.5% of all aneurysms.

It is clear that the response of bifurcation aneurysms following treatment by flow-diverter is understudied and hard to predict. Additionally, the few studies that do exist in the literature often suggest significant limitations. Previous studies have used animal models that rely on surgically created aneurysms, which result in aneurysms with a geometry and thrombosis response that may not be representative of aneurysms that form spontaneously in humans. Clinical studies of patients treated with flow-diverters offer greater sophistication, but the complication rate seen clinically may be biased by the high complexity of the small number of specific cases chosen. Often only the most ‘untreatable’ bifurcation aneurysms (wide-necked, uncoilable, recanalised etc.) are selected for treatment by flow-diverter, as a means of last resort.

Thus, computational fluid dynamics (CFD) techniques present a powerful tool for virtually assessing the effectiveness of flow-diverter treatment on more ‘typical’ human bifurcation aneurysm geometries, cases for which the clinical treatment by flow-diverter would currently present serious ethical concerns. In this study, a number of patient-specific bifurcation aneurysm geometries are virtually treated by flow-diverter. The resulting changes in aneurysm hemodynamic environment due to device deployment are modeled with transient CFD simulations. The shortcomings of such a model are also interrogated. A fundamental limitation to such simulations is the change in outlet boundary condition of a vessel jailed by the flow-diverter device, a condition that cannot be ascertained *a priori*. In this study, a parameterization of the outlet boundary conditions is introduced, to attempt to quantify this error. Finally, a virtual contrast transport model is also discussed, which may pave the way towards *in vivo* verification of CFD simulations and the incorporation of realistic patient-specific boundary conditions. This study builds on work previously discussed by the authors regarding fast deployment algorithms and vessel jailing 22 but focuses specifically on the clinically vital case of bifurcation aneurysm treatment, models such treatment by flow-diverter *in silico* for the first time in the literature, and presents a pathway to future *in vivo* verification.

## Materials and Methods

### Aneurysm Geometries and Virtual Device Deployment

**Figure 1:**
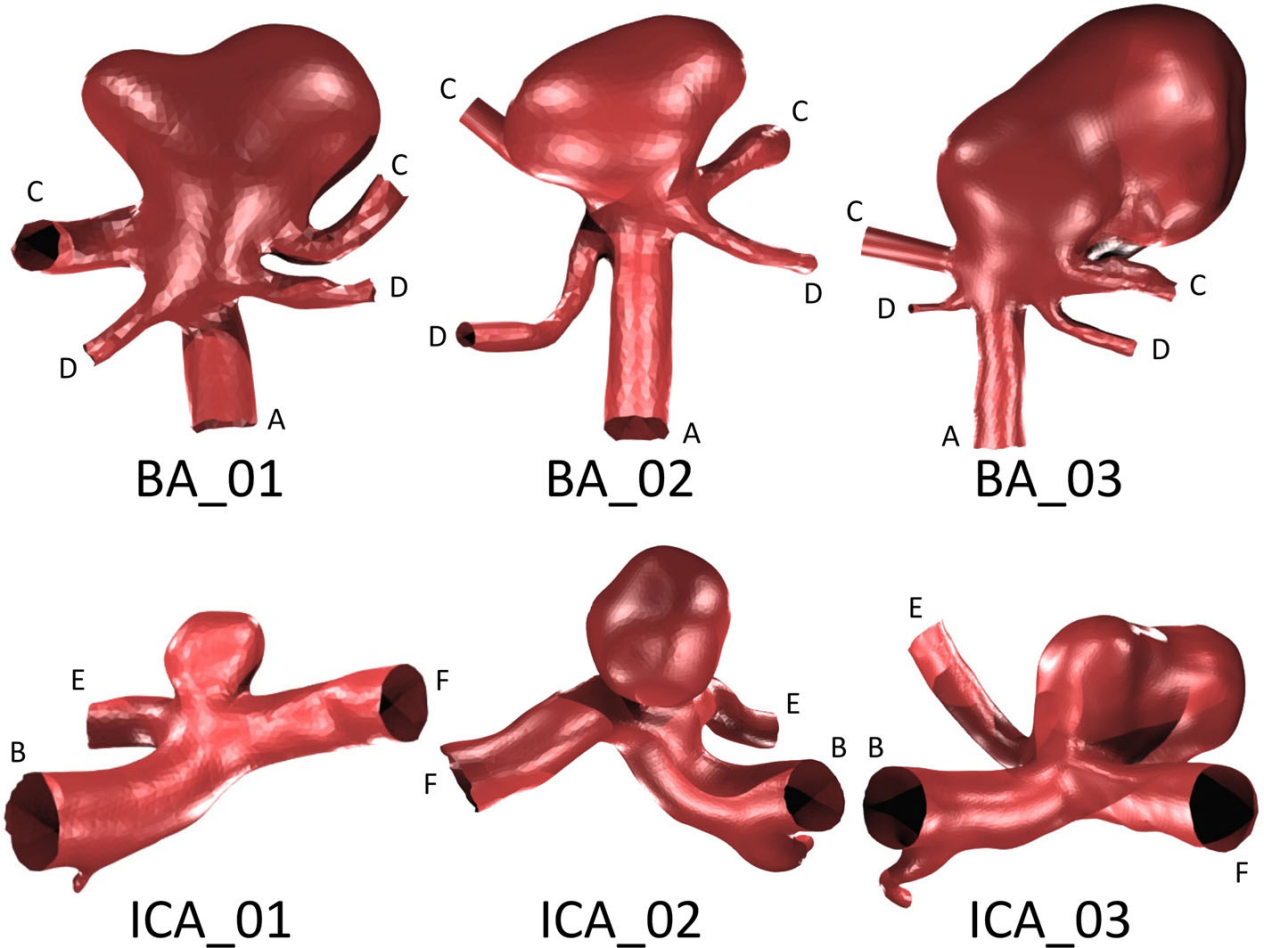
Bifurcation aneurysm geometries selected for virtual flow-diverter deployment. Major arterial segments: (A) basilar artery (BA); (B) internal carotid artery (ICA); (C) posterior cerebral artery (PCA); (D) superior cerebellar artery (SCA); (E) anterior cerebral artery (ACA); (F) middle cerebral artery (MCA). Geometry inlets are located at the BA or ICA respectively (parent vessels) with all remaining arteries (the daughter vessels) corresponding to geometry outlets.

Six anatomically accurate bifurcation aneurysm geometries are selected: three examples of a Basilar tip aneurysm and three examples of an Internal Carotid terminus bifurcation aneurysm. The geometries are segmented from CTA data in OsiriX (OsiriX v.4.1.1, Freeware) and converted to STL format before being imported into Blender (Stichting Blender Foundation, Amsterdam, The Netherlands). The geometries are trimmed to result in parent and daughter vessel lengths of around five vessel diameters distal and proximal to the aneurysm location, as shown in Fig. 1.

The flow-diverter device implanted virtually has a ‘rhomboid’ mesh design with 70% porosity, 48-strand design, a 30 *μ*m wire diameter, and a pore diameter of 120–260 *μ*m. All properties are chosen to approximately match those of commercially available flow-diverter devices: the SILK (Balt Extrusion, Montmorency, France) and the PED (EV3/Covidien, Irvine, CA). The device is created in a range of diameters (2.0–5.0 mm) and lengths (15–40 mm) that are also similar to commercially available devices.^4^ The geometry vessel diameters both proximal and distal to the aneurysms shown in Fig. 1 are measured and an appropriately sized device is chosen in accordance with current device manufacturer’s recommendations. Across all six geometries the flow-diverter devices deployed are of 2.50–3.50 mm diameter and 20–30 mm length.

**Figure 2:**
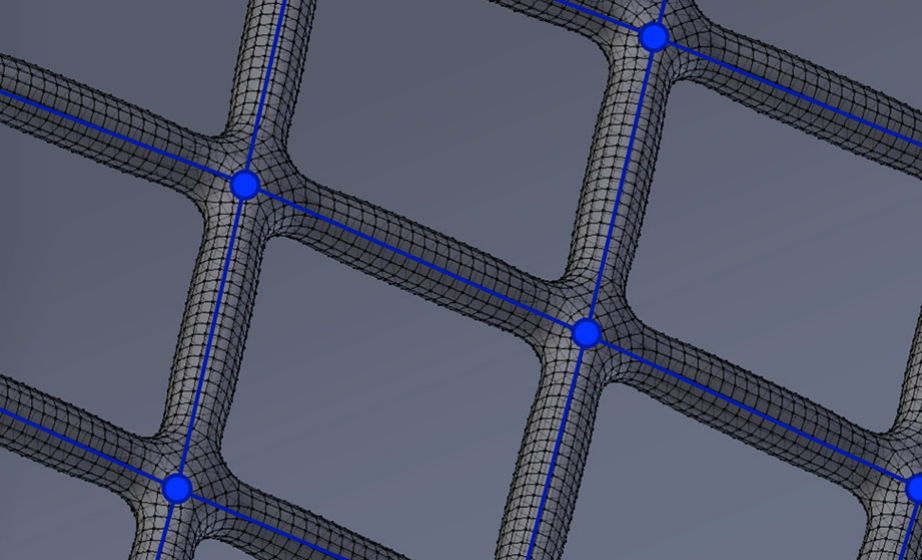
Detail of the centerline vertex representation of the device (blue) and circular cross-section strut thickness added in Blender after deployment (black).

Realistic device deployment is achieved with an in-house fast deployment algorithm that has been discussed in detail by the authors previously.^22^ Briefly, the virtual deployment method is underpinned by a simplified stent representation whereby each strut is approximated by its centerline with no explicit thickness. Deformation of the strut is modeled by both linear and torsional springs connecting each node, with the stiffness of each spring related to the strut thickness and length.^30^,^31^ Contact between the device and vessel wall is modeled with a no-slip condition applied to the device node when the strut makes contact with the vessel wall. Following deployment, the thickness of each device strut is then added back to the strut centerlines by sweeping a cylindrical cross-section in Blender, as shown in Fig. 2.

The speed of the fast deployment method comes at the cost of a number of assumptions, most significantly both linear elasticity and the representation of interwoven wires free to slide over one another as fixed and planar struts. However, errors in deployed position introduced by such assumptions have been shown to be small when compared to other simulations such as full finite element deployments^2^,^30^ as well as *in vitro*^16^ and *in vivo*^5^ experiments.

**Figure 3:**
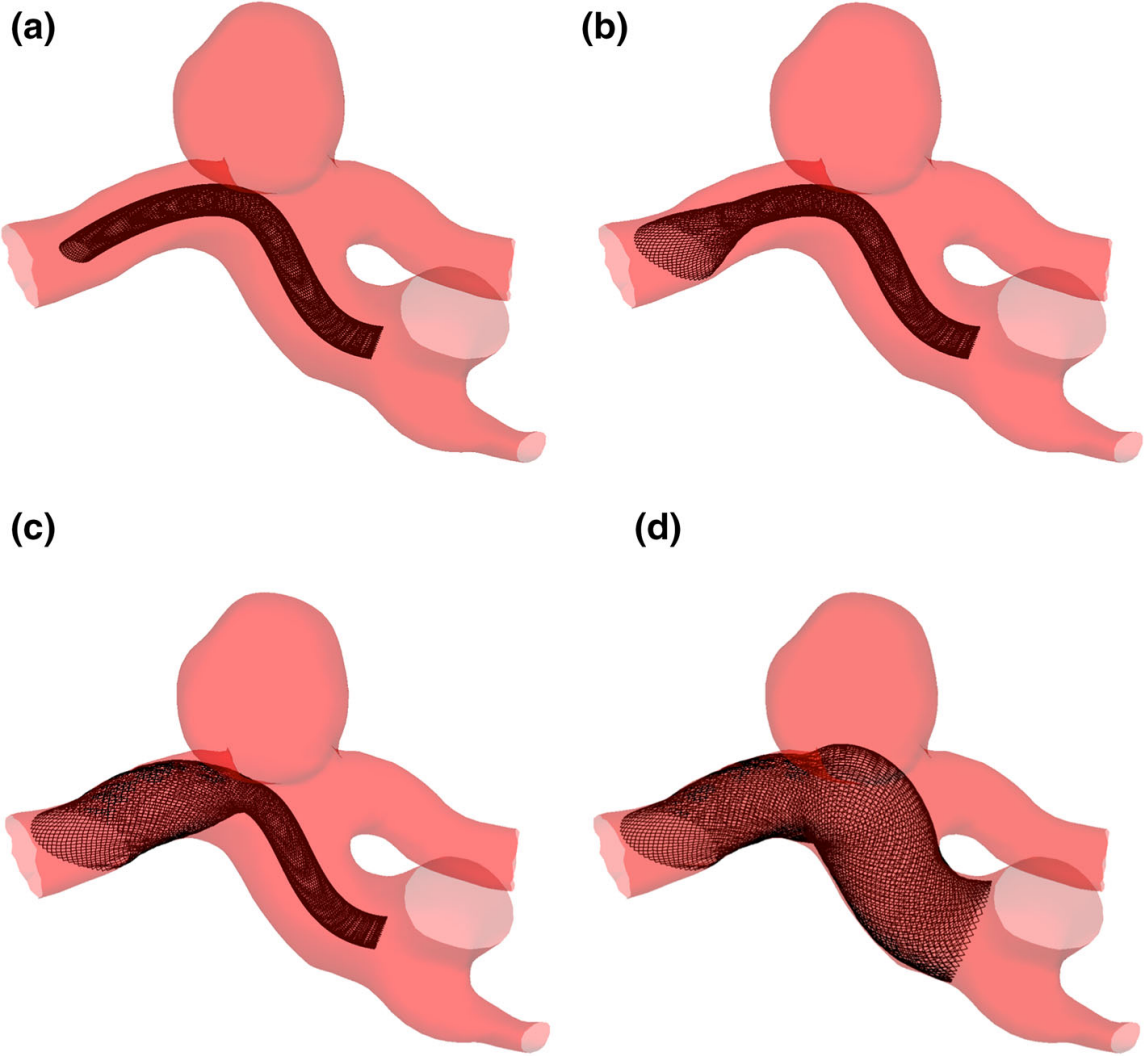
Virtual deployment of a flow-diverter device in the ICA_02 geometry from crimped configuration (a) to deployed position (d).

The initial phase of the deployment process involves crimping the device to simulate the stored elastic energy seen clinically for a flow-diverter compressed in its delivery catheter. In the clinical setting, a flow-diverter is typically deployed with a size 3 or 4 Fr delivery catheter (1–1.33 mm diameter), which results in device compression to around 33% of the original diameter for the flow-diverters virtually deployed in this study. Once crimped to the delivery catheter diameter, the device is virtually aligned to the parent vessel centerline and advanced to the aneurysm neck for expansion, as shown in Fig. 3a.

In order to mimic the gradual device unsheathing seen clinically, the deployment process is initiated at the distal tip of the device and gradually progresses proximally, which results in a deployment similar to that shown in Fig. 3. Due to both the rigid vessel wall and a small degree of device oversizing (as recommended by the manufacturers of devices used clinically) some areas of the device remain under-expanded. This under-expansion is identical to the localized device compression seen in clinical practice, which is common along the inside edge of the device in areas of vasculature with high curvature. In the deployment process across all six aneurysm geometries equilibrium is typically reached in around 200 iterations. Importantly for clinically meaningful rehearsals (often of multiple proposed deployment scenarios), this computation is very fast, approaching real-time speeds. Following device deployment, the mesh of the flow-diverter device is trimmed to remove extreme distal and proximal portions, thereby reducing computational mesh size.

### Computational Modeling

Transient CFD computations are run for each of the six aneurysm geometries both with and without a flow-diverter device deployed. The unsteady 3D Navier–Stokes equations describing blood flow in the geometries are solved assuming incompressible Newtonian flow using the finite volume method in CFD-ACE + (ESI Group, Paris, France). A central difference scheme is used for spatial differentiation as well as a Crank–Nicholson scheme for time marching, both of second order accuracy. A blood density of 1000 kgm^3^ and a dynamic viscosity of 0.004 PaS are assumed. The SIMPLE-Consistent (SIMPLEC) pressure correction method and an algebraic multigrid method for convergence acceleration are used.^20^,^36^,^38^

**Figure 4:**
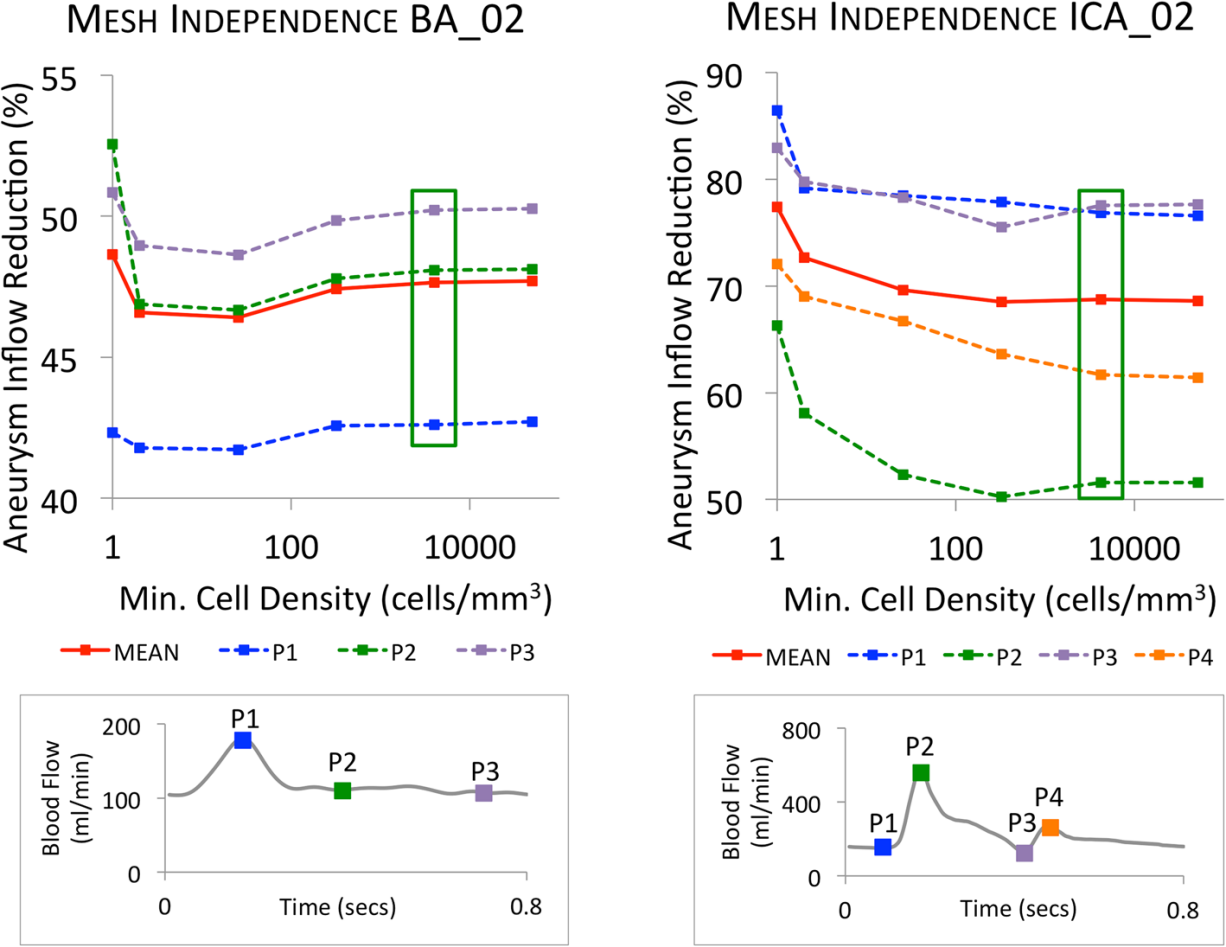
Mesh independence graphs for both the BA_02 and ICA_02 geometries at salient points in the cardiac cycle and mean flow rate. The mesh fineness level at which independence (<1% variation) is reached is indicated in the green box.

A heart rate of 75BPM is assumed with a time step of 0.01 s (see discussion on mesh and time step independence below). The periodic variation of the mean inlet velocity of each parent vessel is scaled to fit volumetric flow curves generated from a 1D model of the arterial tree.^24^ Typical flow profiles for the ICA and BA parent vessels over the cardiac cycle are shown in Fig. 4, with mean flow rates over the cardiac cycle of 230 and 120 mL/min respectively. Such inlet boundary conditions result in a range of instantaneous inlet Reynolds numbers of 169–980 across the geometries, confirming the laminar nature of the flow. A radially-symmetric parabolic inlet velocity profile is prescribed, as the relatively small Womersley number of the inflow (1.68–2.72) and relatively straight portions of vessel upstream of the geometry inlets suggest little departure in velocity profile from a Poiseuille solution.^10^ Three full cardiac cycles are simulated, with the flow distribution only measured in the third cycle in order to remove as many transient effects of the pulsatile flow profile as possible. Aneurysm inflow (and consequently flow reduction) is calculated at each time step through an aneurysm inlet plane meeting the criteria detailed in the following section.

A fixed pressure boundary condition is applied to all outlets of each geometry. It should be noted that such a condition may be unrealistic in the long-term for vessels that are subsequently jailed after stent deployment, when a higher back-pressure is likely due to time-dependent stent endothelialisation and vessel narrowing.^29^ In order to briefly investigate the influence of the outflow pressure boundary conditions applied to both jailed and patent daughter vessels, a number of additional steady state computations are performed. These simulations are conducted at mean parent vessel flow rate and with variable outlet pressure boundary conditions applied to all daughter vessels jailed by the device. Only steady state computations are performed to both represent long-term average conditions and to allow for fixed pressure resistances to be used, eliminating the complexity of the transient flow impedance behavior seen at areas of stenosis, discussed elsewhere in the literature.^37^

This simplified approach to modeling increased resistance in the jailed vessels with increased pressure outlet conditions is of course undermined by no access to such resistance values and no means to easily measure or model them *a priori*. As such, a range of pressure values are chosen, which are of the order of both the pressure gradient present across the vessel bifurcations discussed and that typically induced by a flow-diverter obscuring an aneurysm neck.^15^ The validity of this simplification may be evaluated in the future with *in vivo* imaging of a jailed vessel and a virtual contrast model similar to that discussed in the following section.

Accordingly the outlet pressure boundary conditions of the jailed vessels are increased in 100 Pa increments to a maximum of 300 Pa (2.25 mmHg) above the outlet pressure condition of the stented daughter vessel. Steady state simulations are run with the same meshes and solver parameters as previously discussed. Both sets of transient and steady simulations are run on 16, 2.93 GHz cores with Gigabit interconnectivity and parallelisation implemented with cell-number based domain decomposition.

Finally, in an effort to visualize the aneurysm hemodynamic environment in a more clinically focussed manner, a virtual angiographic contrast model is implemented for one aneurysm geometry. Such a visualization has the potential to become a powerful tool for *in vivo* verification of the predicted *in silico* flow regime, with the predicted rate of aneurysm contrast decay and daughter vessel flow-distributions being compared to clinical results.^32^ Contrast is modeled computationally as an independent scalar quantity introduced at the simulation inlet and transported in the bulk flow in a similar manner to dye particles. Although this method is a major simplification of what is, in reality, a two-fluid mixing problem, the reduction in computational cost of the simplification is considerable. This simplified methodology has been used widely in the literature for over a decade in an attempt to correlate *in vivo* and in silico aneurysm flow patterns, but the use of the technique to virtually model treatment by flow-diverter has thus far been relatively neglected.^6^,^8^,^9^,^27^

The transport of the scalar contrast quantity in the vasculature is governed by the scalar transport equation (Eq. (1)).

1 $$\frac{\partial (\rho \phi )}{\partial t} + \nabla\cdot (\rho \phi \varvec{u}) = \nabla\cdot (\Gamma_{\phi } \nabla \phi ) + S_{\phi }$$

where *φ* = generic scalar quantity, Γ_*φ*_ = the scalar-specific diffusion coefficient and S_*φ*_ = any scalar source terms.

The scalar quantity does not alter the simulated blood density or viscosity. The scalar has a diffusivity in blood of 1.0 × 10^−7^ m^2^/s, which is an order of magnitude estimate based on the diffusivity of tracer particles in blood flow.^25^ Additionally, the dominance of convection over diffusion in the flow is likely to render the effects of any inaccuracy in estimating the diffusivity small.^28^

A uniform contrast bolus with a value of unity over the entire cardiac cycle is applied at the geometry inlet, with the resulting contrast residence profile assumed to represent typical contrast residence averaged over one cardiac cycle. The contrast bolus is introduced after two full cardiac cycles (1.60 s real time) to reduce the influence of initial transient flow effects. The simulation is run for a further two cardiac cycles after the introduction of the contrast ceases, giving a total simulation time of five cardiac cycles (4.00 s real time). The model is implemented in the CFD-ACE + platform (ESI Group, Paris, France) with all simulations run on 32, 2.93 GHz cores and the same hardware architecture previously detailed.

### Mesh Independence

Transient flow mesh independence tests are performed for representative geometries from each location (in this case, geometries BA_02 and ICA_02), with and without a device deployed. The grid independence test metric employed is the mean volumetric flow at the aneurysm neck. The meshing of each geometry is completed with a Projected Single Domain con-conforming mesh, an Omnitree Cartesian tree type, and three near-wall Cartesian layers to give a smooth and well-resolved boundary definition. Meshes are generated at increasing levels of fineness with minimum cell densities in the range of 1–50,000 elements/mm^3^. Aneurysm inflow is measured through a plane defined at the aneurysm neck. The plane must be placed as close to the natural neck of the aneurysm without falling too close to the device, which can lead to spurious results from local vorticity, as observed by Kim *et al.*^13^ The solution is assumed mesh independent when the discrepancy in aneurysm inflow between two consecutive meshes falls below 1%. Aneurysm inflow is measured at salient points in the cardiac cycle (peak systole, dicrotic notch etc.) as well as a mean aneurysm inflow calculated over a single cardiac cycle. Three full cardiac cycles were simulated with results measured from the final cycle in order to remove transient effects.

Simulations run for both representative geometries suggest mesh independence at meshes with a typical minimum cell density finer than 4000 elements/mm^3^, as shown in Fig. 4. Such mesh densities meet the requirements discussed in the literature for smooth velocity and WSS resolution 33,34 and are similar to the mesh-independent values calculated for cerebral aneurysms both with and without devices deployed previously obtained by the authors.^21^,^22^ Additionally, at this level, the number of mesh elements in each geometry measurement plane exceeds the recommendation of Jou *et al.* that is required to fully resolve flow features.^12^ This mesh independence analysis results in mesh sizes that vary between 3,410,000 and 7,200,000 elements across all six geometries, both with and without the device implanted.

Mesh independence tests were also performed at the same mesh resolution levels but using a metric of variation in wall shear stress (WSS). Less than 1% variation in spatio-temporal mean and maximum aneurysm WSS was seen at the same mesh resolution highlighted in Fig. 4 (>4000 elements/mm^3^). WSS measurements are considerably more sensitive to local mesh resolution (as a derivative of the velocity field) than inflow (the integral of the velocity field), however, aneurysm inflow was chosen as a metric in this study in order to also capture the importance of mesh refinement at the aneurysm neck and local to the device struts, where the magnitude of velocity gradients often far exceed those at the aneurysm wall.

The independence of the transient flow solution with time-step size is also investigated. Simulations of the representative geometries were run at time-steps (Dt) of 0.05, 0.01 and 0.005 s. Results indicated that Dt = 0.01 s offered both good convergence of solution (unlike Dt = 0.05 s) and no significant change in predicted flow pattern when compared to Dt = 0.005 s. Such a choice of time-step is consistent with similar CFD studies.^12^,^17^,^35^

## Results

**Figure 5:**
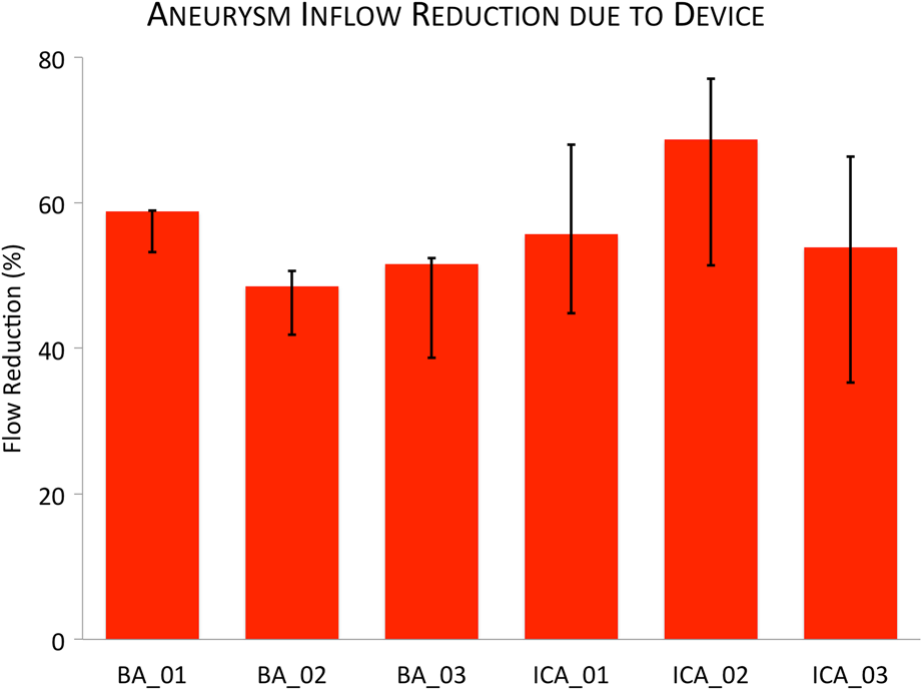
Mean aneurysm inflow reduction due to device deployment. The range in flow reduction seen over the cardiac cycle is indicated in black.

**Figure 6:**
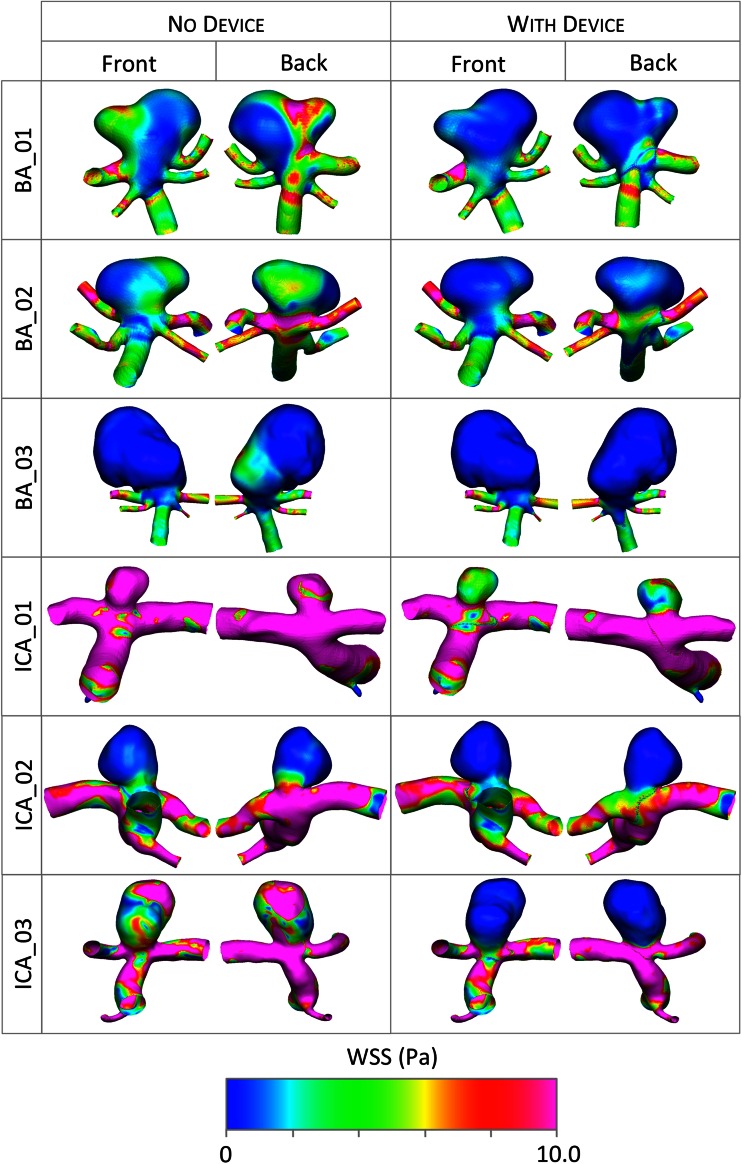
WSS at peak parent vessel flow rate both before and after device deployment.

**Figure 7:**
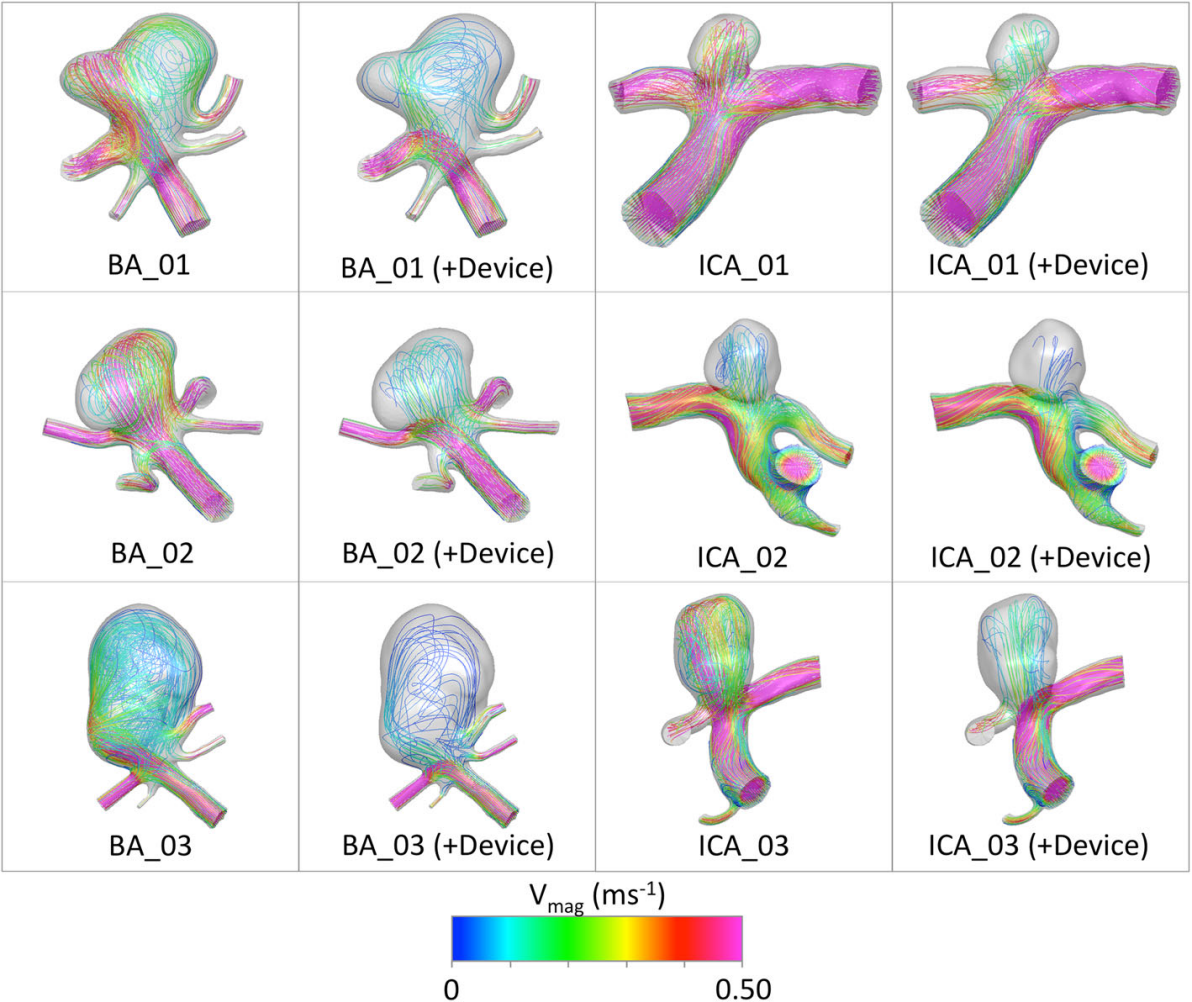
Lines tangent to instantaneous velocity vectors, at mean parent vessel flow rate, for all six aneurysm geometries with and without a device deployed. Larger variation in aneurysm flow pattern (jet location, number and location of areas of circulation etc.) after device deployment appears to correspond to greater WSS variation, shown in Fig. 6.

In the transient simulations with and without a device deployed, each time step for all aneurysm geometries converges to five orders of magnitude in less than 100 iterations and around 45 min for a given time step. The aneurysm inflow reduction due to flow-diverter deployment is shown in Fig. 5 as both the average over the entire cardiac cycle and the range of instantaneous reductions observed. The percentage aneurysm inflow reduction is calculated from the relative inflow rate reduction (inflow without device - inflow with device) divided by the original inflow rate (without device). Wall shear stress (WSS) at peak parent vessel flow rate, both with and without the flow-diverter device deployed, is shown in Fig. 6. Flow streamlines at mean parent vessel flow rate before and after virtual treatment are shown in Fig. 7. The stented daughter-vessel (mean) flow rates, both before and after device deployment, are summarized in Table 1.

**Table 1:** Stented daughter-vessel mean flow rate with and without a flow-diverter device deployed.

	BA_01	BA_02	BA_03	ICA_01	ICA_02	ICA_03
No device (mL/min)	62.2	37.6	64.4	162	155	165
With device (mL/min)	63.0	35.6	64.4	166	154	166
Change (%)	+1.29	−5.32	0.00	+2.47	−0.65	+0.60

**Figure 8:**
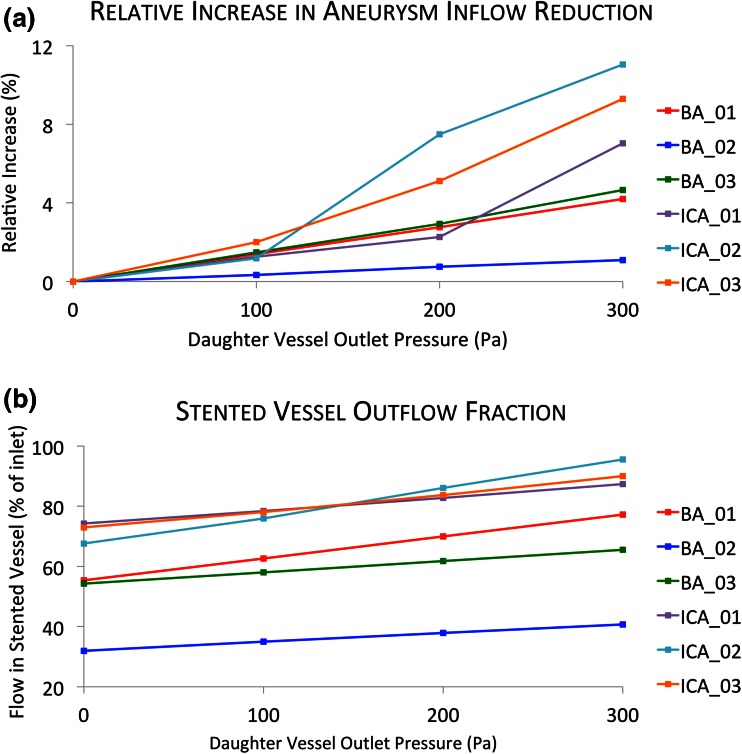
The effect of jailed daughter vessel outlet pressure conditions (+0 to 300 Pa) on aneurysm inflow reduction due to the device (a) and stented daughter vessel outflow distributions (b).

The steady state simulations, run with parameterized increasing jailed daughter vessel outlet pressure conditions as discussed above, all converge to five orders of magnitude in less than 2000 iterations and around 12 h per simulation. The relative increase in device-induced aneurysm inflow reduction as a result of the change in outlet pressure condition is plotted in Fig. 8. The fraction of total flow exiting the daughter vessel implanted with the device is also shown in Fig. 8, for the range of outlet pressure conditions imposed. The corresponding sum of flows, leaving the jailed daughter vessel(s) with a 0 Pa and +300 Pa outlet pressure boundary condition, are summarized in Table 2.

**Table 2:** Total jailed vessel(s) outflow in each geometry with an unaltered outlet pressure (0 Pa) and the maximum increased outlet pressure (+300 Pa).

	BA_01	BA_02	BA_03	ICA_01	ICA_02	ICA_03
0 Pa (mL/min)	57.8	82.4	55.6	68.0	75.0	65.0
+300 Pa (mL/min)	29.5	71.8	41.9	33.4	10.3	24.0
Change (%)	−49.0	−12.9	−24.6	−50.9	−86.3	−63.1

**Figure 9:**
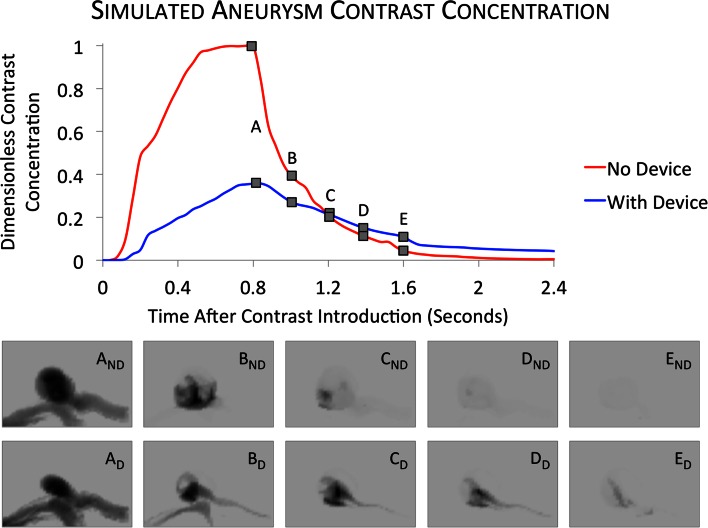
Simulated aneurysm contrast residence curves and virtual angiography generated for the ICA_02 geometry both with and without a flow-diverter device deployed.

Finally, the transient simulation of the ICA_02 geometry modeling virtual contrast transport is completed, with each simulation time-step converging to five orders of magnitude residual reduction in less than 100 iterations and in a typical solution time of 30 min per time step. The corresponding relative contrast concentration (mean concentration over the entire aneurysm dome) for the geometry with and without a flow-diverter device deployed is plotted in Fig. 9. A number of ‘virtual angiograms’ are generated by projecting the 3D contrast concentration into a 2D plane, and are also shown in Fig. 9.

## Discussion

### Aneurysm Inflow Reduction and Wall Shear Stress

The reduction in aneurysm inflow rate after flow-diverter deployment is shown as both a mean over the cardiac cycle and the range of values seen over the cardiac cycle in Fig. 5. The larger range in ICA parent vessel flow rate compared to the BA (a range of 130–570 and 105–180 mL/min respectively) results in a greater range of aneurysm inflow reduction seen over the cardiac cycle in the ICA geometries. Despite the distinct divide in the range of flow reductions between the two aneurysm locations, the mean flow reduction seen across all six aneurysm geometries remains relatively constant and falls within the range of around 50–70%.

The action of the flow-diverter device appears to be to diffuse flow entering the aneurysm dome and to remove strong inflow jets. This reduction in jetting behavior can be clearly seen in the WSS plots of the aneurysm geometries both with and without a device deployed, which are shown in Fig. 6. Figure 6 depicts the WSS at peak systole, and although aneurysm WSS magnitude is seen to vary throughout the cardiac cycle, the broad pattern WSS distribution remains very similar to the peak-systole distributions shown in the figure.

Across five of the six aneurysm geometries simulated (all but ICA_01) the peak values of WSS within the aneurysm are reduced to an approximately normal range of less than 4 Pa, after flow-diverter deployment.^18^ Although the peak WSS remains higher than 4 Pa in the ICA_01 geometry after device deployment, the relative reduction in WSS due to the device is still substantial. Neither the degree of overall WSS reduction due to the device nor the persistence of localized aneurysm WSS peaks following device deployment appears to correlate with the aneurysm inflow reduction achieved by the flow-diverter. For example, the ICA_02 case shows very little change in aneurysm WSS pattern or magnitude after treatment, with a 68.7% flow reduction. By contrast, the ICA_03 case shows complete obliteration of strong WSS peaks and a large overall reduction in WSS following device deployment, but a slightly lower inflow reduction of 53.9%. A possible explanation for this discrepancy is seen in Fig. 7, where the more dramatic changes in WSS reduction are seen to correspond to geometries with greater changes in aneurysm flow pattern (inlet jet location, number and locations of areas of circulation etc.) after device deployment, such as the BA_01, ICA_01 and ICA_03 cases.

### The Effect of Daughter Vessel Jailing

Table 1 shows very little change in stented daughter vessel flow rate after flow-diverter deployment (<5% in 5/6 cases and <1% in 3/6 cases). These results suggest that the presence of the flow-diverter alone (the outlet pressure of all daughter vessels remain constant) does not substantially alter the distribution of flow to each daughter vessel across all six aneurysm geometries.

Figure 8a suggests that the relative change in mean aneurysm inflow reduction after increasing the daughter vessel outlet pressure conditions is also small; in all cases less than an 11% increase in device-induced inflow reduction is seen at the maximum outlet pressure of +300 Pa. Even in the most extreme case of the ICA_02 geometry, where the +300 Pa pressure condition results in near complete occlusion of the jailed vessel, comparatively little change in aneurysm inflow is seen.

The overall pressure gradient (inlet–outlet) across the six geometries after device deployment with constant daughter vessel outlet pressures is in the range of 144–528 Pa. Consequently, the maximum pressure bias (+300 Pa) applied at the jailed daughter vessels represents a relative pressure increase of ≈50–200% of the overall stented geometry pressure gradient, depending upon the aneurysm geometry. More dramatic changes in aneurysm inflow reduction (Fig. 8a) are seen in geometries where the increased daughter vessel outlet pressure is large relative to the overall pressure gradient for the geometry. Greater increases in aneurysm inflow reduction are seen in the ICA cases (7.0–11.0%), where the maximum boundary-condition increase (+300 Pa) represents 79–208% of the overall geometry pressure gradient. Lower increases in inflow reduction are seen in the BA cases (1.1–4.7%), where the maximum boundary-condition increase represents 57–111% of the overall geometry pressure gradient.

Figure 8b shows a greater fraction of the total outflow exiting through the stented daughter vessel across all three ICA cases than for the BA geometries. Such a pattern is most likely due to the reduced number of daughter vessels in the ICA geometries (two instead of the four vessels in the BA geometries), which results in the stented vessel occupying a larger fraction of the total outlet area in the ICA case. All three ICA geometries show relatively similar fractions of outflow in the stented vessel at each outlet pressure condition, ranging from 68 to 73% with a 0 Pa outlet condition and 87–96% with a +300 Pa jailed vessel outlet pressure condition. By contrast, substantial variation in the fraction of flow leaving the stented vessel is seen in the three BA cases with the fraction ranging from 32 to 54% with a 0 Pa outlet condition and 41–77% with a +300 Pa jailed vessel outlet pressure condition. This greater variation seen in the BA cases is attributed to greater localized geometric variations; more variation is seen for the BA cases, where anatomical differences (relative diameters, patency, and positions of daughter vessels) are more substantial across the geometries. Additionally, such variation also suggests that average models of the vasculature may not be effective for outflow boundary condition estimation.

From Fig. 8b, it is also clear that the fraction of flow leaving the stented daughter vessel in each geometry appears to scale approximately linearly with the increased outlet pressure of the jailed daughter vessels. However, such a linear correlation must breakdown as the increasing pressure condition of the jailed daughter vessels moves towards directing 100% of the flow through the stented daughter vessel (complete occlusion of the jailed daughter vessels). The decrease in jailed daughter vessel outflow due to the increased outlet pressure boundary conditions is summarized in Table 2. From the table, it is apparent that while aneurysm inflow reduction is relatively insensitive to outflow pressure increases, substantial reductions in jailed vessel flow rate are seen after applying the +300 Pa boundary condition. Across all six geometries, jailed daughter vessel flow falls by 12.9–86.3% with the 300 Pa pressure increase. As previously discussed, the greatest reductions are seen for geometries where the overall pressure gradient for the geometry (inlet–outlet) is small compared to the 300 Pa pressure increase. The range of the pressure-induced reduction in jailed vessel flow rate seen in Table 2 is significant and varies from almost no change (12.9% reduction) to near-complete flow stasis (86.3%). Such sensitivity again illustrates the highly patient-specific nature of the flow patterns seen within the simulated vasculature and suggests that entirely patent-specific boundary conditions may be necessary to accurately model flow-diverter treatment outcomes in bifurcation aneurysms.

### Virtual Angiography and *In Vivo* Verification

Finally, the results of the two virtual contrast simulations for the ICA_02 geometry are shown in Fig. 9 as both contrast residence curves and virtual angiography. For simplicity, these simulations are conducted assuming equal outlet pressures for both patent and jailed daughter vessels. The results of the ICA_02 case serve as an illustrative example for how future studies may begin to verify simulations with *in vivo* results. Such verification would offer particular insight into the bifurcation aneurysm cases discussed here, where currently the lack of accurate *a priori* boundary conditions for vessels jailed by a flow-diverter severely limits the applicability of any simulation results.

From Fig. 9, it is clear that device deployment substantially alters the distribution and decay of contrast in the aneurysm dome. The degree of contrast infiltration is markedly reduced after device deployment (compare angiographic images A_ND_ and A_D_ in Fig. 9), which supports the predicted reduction in both aneurysm inflow rate and inflow jetting seen previously. The mechanism of clearing the contrast from the aneurysm dome also appears substantially altered after device deployment: with no device deployed, contrast washes out of the aneurysm symmetrically with both sides of the aneurysm dome visible throughout the decay. After device deployment, the decay appears more asymmetric with slow-moving flow in the jailed vessel gently carrying contrast away from the aneurysm dome at a reduced rate.

The decay portions of the curves in Fig. 9 are well described by an exponential decay curve, when fitted to relative contrast concentrations ranging from 100% to 10% of the peak concentration (coefficient of determination (*R*^2^) >0.90). Around a 67% reduction in decay rate is seen after device deployment with a ‘No Device’ exponential decay rate of 3.86 s^−1^ and a `with device’ decay rate of 1.46 s^−1^. It should be remembered, however, that these results along with those of the previous section are currently entirely unverified.

If *in vivo* angiography of the ICA_02 geometry both before and after flow-diverter treatment had been available to the authors, the contrast decay rate and the fraction of contrast leaving each daughter vessel could have been used to approximately verify the simulation outlet boundary conditions imposed. This would then allow for patient-specific boundary conditions of the *in vivo* geometry to be estimated indirectly. However, the computational power required for such a study is considerable, as a number of virtual contrast simulations would need to be implemented at varying outlet pressure conditions. Finally, such a methodology would only be viable for bifurcation aneurysms treated by flow-diverter alone with no coils present in the aneurysm dome.

## Conclusions

This computational study of six cerebral bifurcation aneurysms has shown that substantial reductions in aneurysm inflow (mean reduction >50%) may be achieved with treatment by flow-diverter. Flow-diverter deployment also reduced both peak and mean WSS values within the aneurysm dome; in five aneurysm geometries the aneurysm WSS distribution throughout the cardiac cycle was reduced to values considered physiologically normal (< 4 Pa). Assuming no change in jailed vessel pressure boundary conditions, the deployment of a flow-diverter device in all six aneurysms does not substantially alter the distribution of flow to the rest of the arterial tree.

Simulations conducted with increased outlet pressure boundary conditions for daughter vessels jailed by a flow-diverter device showed little variation in aneurysm inflow reduction: the maximum 300 Pa (2.25 mmHg) increase in daughter vessel outlet pressure resulted in less than 11% increase in flow-diverter induced aneurysm inflow reduction. However, the increase in jailed vessel outlet pressure does have a dramatic effect on daughter vessel flow rate: an increase of 300 Pa in outlet pressure was shown to result in a 12.9–86.3% reduction in the total daughter vessel mean flow. Such sensitivity of flow rate to outlet pressure condition reinforces the highly patient-specific nature the flow patterns seen. However, these results are entirely unverified and further development of *in vivo* verification techniques will be needed to begin to investigate the validity of the boundary conditions imposed in such simulations.

Finally, the modeling of angiographic contrast transport within the aneurysm geometry, both with and without a flow-diverter device deployed, has been discussed. Simulated contrast decay in the simulated aneurysm is well described by an exponential decay curve, which, in the case discussed (ICA_02), results in a 67% reduction in contrast decay rate after flow-diverter deployment. Finally, the incorporation of virtual contrast residence models into future patient-specific aneurysm CFD simulations and combined with clinical angiography, may provide a viable route to *in vivo* verification of simulation boundary conditions and flow patterns.
